# Supplementary material for: The influence of TC0668 on glycometabolism modulation in Chlamydia muridarum-infected host cells
Source: Microbiol Spectr. 2025 Oct 13;13(11):e03051-24. doi: 10.1128/spectrum.03051-24 (PMC12584682; doi:10.1128/spectrum.03051-24)
Supplement: Figures S1 and S2 — Figure S1: Schematic representation of cellular glucose metabolism, encompassing glycolysis and oxidative phosphorylation. Figure S2: The PI3K inhibitor LY294002 exerts no effect on the growth or development of Cm TC0668wt and TC0668mut strains. [file spectrum.03051-24-s0001.docx]

**SUPPLEMENTAL INFORMATION**

**
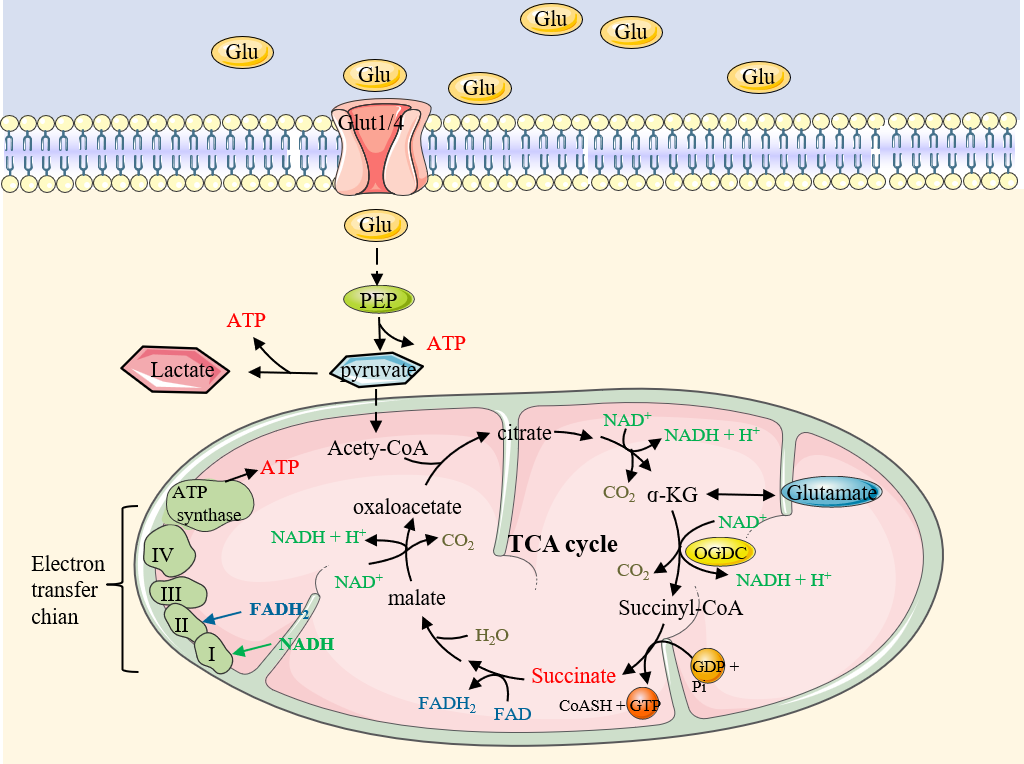
Figure S1.** Schematic representation of cellular glucose metabolism, encompassing glycolysis and oxidative phosphorylation.


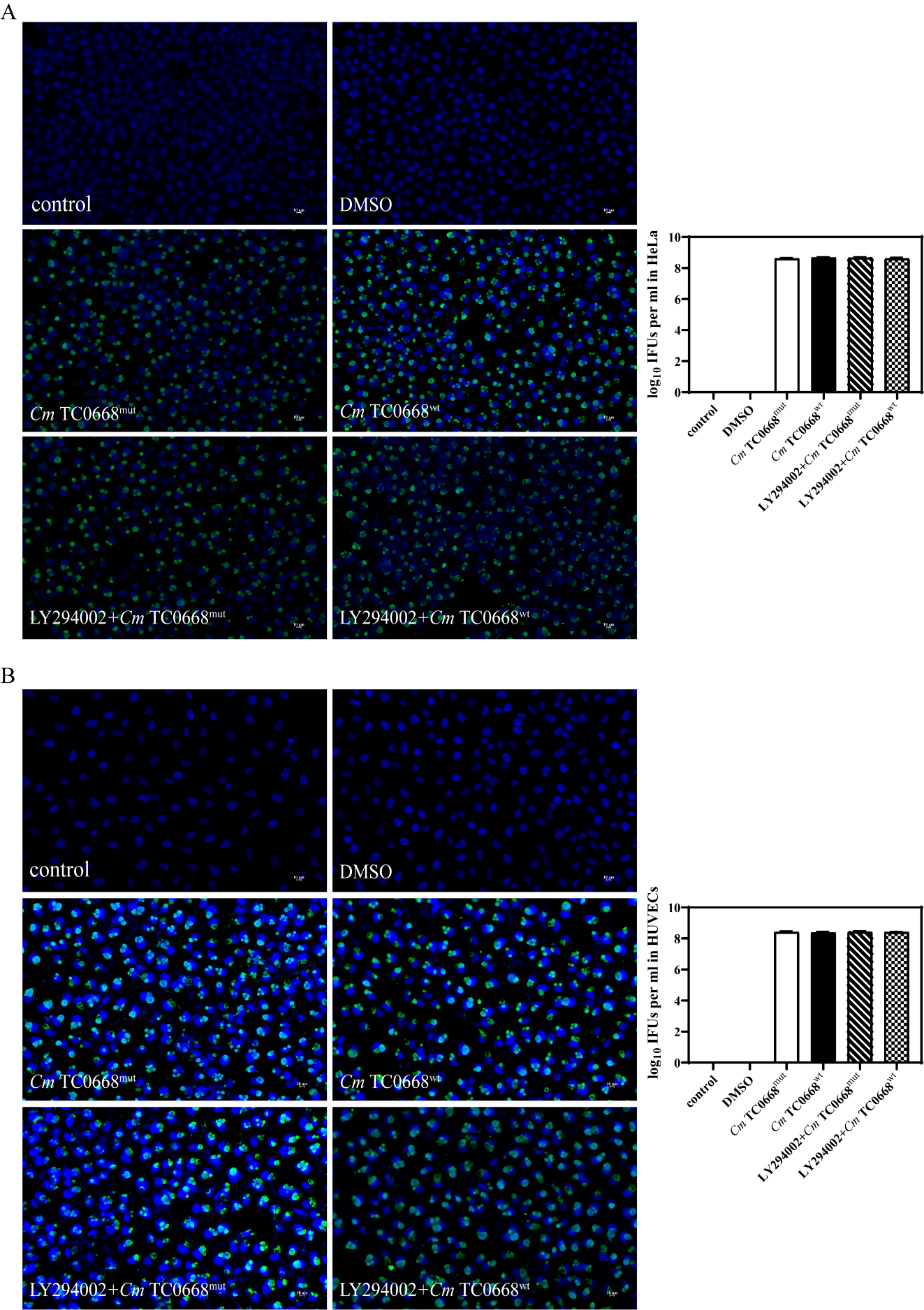


**Figure S2.** The PI3K inhibitor LY294002 exerts no effect on the growth or development of *Cm* TC0668^wt^ and TC0668^mut^ strains. Chlamydial inclusion bodies (green) are visible in both *Cm* TC0668^wt^ or TC0668^mut^-infected cells. Magnification, ×200. (A) HeLa cells were pretreated with 20 µM PI3K inhibitor LY294002 for 1 hour**,** infected with *Cm* TC0668^wt^ or TC0668^mut^ strains for 18 hours, and inclusion forming units (IFUs) were detected by indirect immunofluorescence assay (IFA). (B) HUVECs were pretreated with PI3K inhibitor at a concentration of 20 µM for 1 hour, and infected with *Cm* TC0668^wt^ or TC0668^mut^ strain for 42 hours. The IFUs were measured by IFA.
